# Orosensory Perception of Fat/Sweet Stimuli and Appetite-Regulating Peptides before and after Sleeve Gastrectomy or Gastric Bypass in Adult Women with Obesity

**DOI:** 10.3390/nu13030878

**Published:** 2021-03-08

**Authors:** Arnaud Bernard, Johanne Le Beyec-Le Bihan, Loredana Radoi, Muriel Coupaye, Ouidad Sami, Nathalie Casanova, Cédric Le May, Xavier Collet, Pascaline Delaby, Cindy Le Bourgot, Philippe Besnard, Séverine Ledoux

**Affiliations:** 1 UMR Lipides/Nutrition/Cancer 1231 INSERM/AgroSup Dijon/Univ. Bourgogne-Franche Comté, 21000 Dijon, France; arnaud.bernard@u-bourgogne.fr; 2 UF de Génétique de l’Obésité et des Dyslipidémies, Service de Biochimie Endocrinienne et Oncologique, Centre de Génétique Moléculaire et Chromosomique, Groupe Hospitalier Pitié-Salpêtrière (APHP), 75013 Paris, France; johanne.lebihan@aphp.fr; 3 Fonctions Gastro-Intestinales, Métaboliques et Physiopathologies Nutritionnelles Inserm UMR1149, Centre de Recherche sur l’Inflammation Paris Montmartre, 75018 Paris, France; 4 Service d’odontologie, Hôpital Louis Mourier (APHP), 92700 Colombes, France; loredana.radoi@lmr.aphp.fr; 5 Explorations Fonctionnelles, Hôpital Louis Mourier (APHP), Université de Paris, 92700 Colombes, France; muriel.coupaye@aphp.fr (M.C.); ouidad.sami@aphp.fr (O.S.); nathalie.casanova@aphp.fr (N.C.); 6 UMR 1087 INSERM/6291 CNRS, 44007 Nantes, France; Cedric.Lemay@univ-nantes.fr; 7 UMR 1048 INSERM/Toulouse III, 31400 Toulouse, France; xavier.collet@inserm.fr; 8 Lesieur Groupe Avril, 92600 Asnières-sur-Seine, France; pdelaby@lesieur.fr; 9 Tereos, 77230 Moussy-le-Vieux, France; cindy.lebourgot@tereos.com; 10 Physiologie de la Nutrition, Agrosup Dijon, 26, Bd Dr Petitjean, 21000 Dijon, France

**Keywords:** fat taste, sweet taste, obesity, bariatric surgery, appetite-regulating hormones, health

## Abstract

The aim of this study was to explore the impact of bariatric surgery on fat and sweet taste perceptions and to determine the possible correlations with gut appetite-regulating peptides and subjective food sensations. Women suffering from severe obesity (BMI > 35 kg/m^2^) were studied 2 weeks before and 6 months after a vertical sleeve gastrectomy (VSG, *n* = 32) or a Roux-en-Y gastric bypass (RYGB, *n* = 12). Linoleic acid (LA) and sucrose perception thresholds were determined using the three-alternative forced-choice procedure, gut hormones were assayed before and after a test meal and subjective changes in oral food sensations were self-reported using a standardized questionnaire. Despite a global positive effect of both surgeries on the reported gustatory sensations, a change in the taste sensitivity was only found after RYGB for LA. However, the fat and sweet taste perceptions were not homogenous between patients who underwent the same surgery procedure, suggesting the existence of two subgroups: patients with and without taste improvement. These gustatory changes were not correlated to the surgery-mediated modifications of the main gut appetite-regulating hormones. Collectively these data highlight the complexity of relationships between bariatric surgery and taste sensitivity and suggest that VSG and RYGB might impact the fatty taste perception differently.

## 1. Introduction

Ubiquitous availability of tasty energy-dense foods rich in fat and sugar is thought to play a significant role in the prevalence of obesity worldwide [1]. Interestingly, a preferential consumption of highly palatable foods (i.e., pleasant in the mouth) is often observed in obese subjects [2,3,4], suggesting that the perception of orosensory sensations in response to fat and sweet stimuli might be compromised in obesity leading to unhealthy food choices.

Bariatric surgery is the most efficient method to reduce severe obesity and improve associated comorbidities (e.g., type-2 diabetes). To date, Roux-en-Y gastric bypass (RYGB) and vertical sleeve gastrectomy (VSG) are commonly performed procedures by reason of their long-term effectiveness [5], despite important anatomic differences [6]. Paradoxically, how these surgery procedures work is not yet fully understood, likely due to the complexity of induced homeostatic changes. For example, the beneficial effect of RYGB on the remission of type-2 diabetes appears to be not fully attributable to weight loss, as previously expected [7,8].

Gustation, known to be a key determinant for food selection, is a complex phenomenon including the oral detection of sensory inputs, central identification of tastants, evaluation of their intensity and generation of hedonic adaptations contributing to the decision to eat or not [9]. A growing number of studies suggests that taste perception might be affected by bariatric surgery [10,11,12]. Nevertheless, whether bariatric surgery-induced change of gustatory sensitivity is a plausible contributor to a successful weight loss is still elusive. Indeed, the analysis of both subjective orosensory sensations and ingestive behavior after bariatric surgery usually shows an improvement of the orosensory perception of fat and sweet stimuli associated with a decreased interest for and intake of high caloric foods [12]. However, the fact that these data are generated by using indirect methods (e.g., self-reported questionnaires) limits their physiological relevance [12]. Although direct sensory methods (e.g., alternative force-choice tests, taste strips) were used to explore the changes of gustatory sensitivity (mainly sweet taste) in patients with obesity, they led to discrepant results, with some studies reporting a lower sweet threshold after surgery (i.e., a better sensitivity), [13,14,15], while others showed no change [16,17,18,19,20,21]. Moreover, despite the well-known high palatability, and in turn attractivity, of fat-rich foods [22], we do not know whether the fat taste sensitivity is modified after bariatric surgery and what the respective impacts of RYGB and VSG procedures are on this sensory parameter. Finally, the fact that RYGB and VSG differentially alter the secretion pattern of appetite-regulating peptides (i.e., insulin, glucagon-like-peptide-1 (GLP-1), peptide YY (PYY) and ghrelin) [23], known to be involved in modulating taste perception [24], raises an additive question: does the global change of gut hormone balance induced by bariatric surgery affect fatty and sweet taste sensitivity?

To explore these issues, oral perception thresholds in response to fat and sweet stimuli, subjective changes of food sensations and postprandial levels of appetite-regulating hormones were analyzed before and 6 months after VSG or RYGB surgery in adult women suffering from severe obesity.

## 2. Subjects and Methods

### 2.1. Subjects

This clinical study was approved by a French ethics committees (Comité de Protection des Personnes, CPP n° 15-032 and Agence Nationale de la Sécurité des Médicaments, ANSM n° 150811B-21) and was registered at Clinical Trials (NCT#02497274). Severe obese (BMI ≥ 35 kg/m^2^) adult (≥18 and ≤55 y-old, mean age = 38.4 ± 1.3 y) patients who were candidates for bariatric surgery were selected according to the following exclusion criteria: medications known to alter taste, buccal decays, previous surgical treatment of obesity, diabetes (hypoglycemic drug intake or fasted plasma glucose level >7 mmol/L), chronic inflammatory diseases and tobacco use. All subjects received detailed information about the study and provided a written consent. To avoid gender-mediated bias [25], this study was conducted on women only.

**Figure 1:**
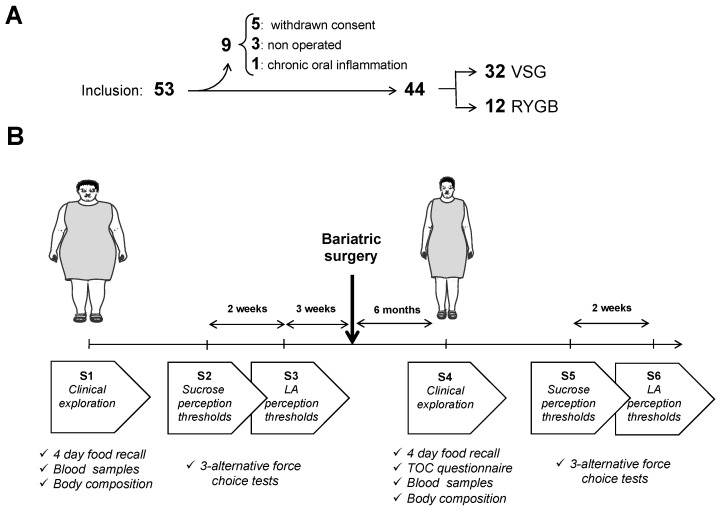
Cohort composition and study design. (**A**) From the 53 patients initially selected, 44 were included, 32 underwent a vertical sleeve gastrectomy (VSG) and 12 a Roux-en-Y gastric bypass (RYGB). (**B**) Before surgery, patients were subjected to 3 successive sessions (S1–S3) including a clinical exploration and the determination of sucrose and linoleic acid (LA) perception thresholds. The same exploratory design was used 6 months after surgery.

On the 53 subjects initially included, 9 subjects were secondary excluded: 3 were not operated, 5 withdrew their consent due to constraints of repetitive visits and 1 was not included due a chronic oral inflammation (Figure 1A). Among the 44 selected patients, 32 underwent a VSG procedure and 12 an RYGB one. All the interventions were performed laparoscopically, as previously described [26], with a 150 cm alimentary limb and a 60 cm biliopancreatic limb for RYGB according to international recommendations [27]. Pre- and postoperative multidisciplinary managements were performed in the Louis Mourier Hospital (Assistance Publique Hôpitaux de Paris—APHP) and have been previously described elsewhere [28].

Since the lipid perception threshold appears to be unrelated to BMI in humans [29] and consistent with the postsurgery timing of previous studies [14,15,16,19,30], while limiting the risk of patients leaving the study, the postoperative time period chosen in this protocol was 6 months. Each patient was subjected to a set of 3 successive sessions before (S1 to S3) and 6 months after (S4 to S6) surgery (Figure 1B). Sessions 1 and 4 (S1/S4) included a dietary habit questionnaire (4-day food recall), a blood collection and an analysis of the body composition including body fat mass by multifrequency impedancemetry (Secca, France). A questionnaire on the taste and olfaction changes (TOCs) [31], adapted to include questions about fat-mediated sensations, was added to S4 (i.e., the first postoperative session). An analogic scale scored from 1 (no change) to 10 (maximal change) was used to evaluate the subjective gustatory and olfactory changes. Determination of fat and sweet taste changes was performed by using the 3-alternative force-choice procedure (3-AFC) [32] during sessions S2/S3 and S5/S6, respectively.

### 2.2. 3-Alternative Force-Choice Tests

LA and sucrose were used to evaluate the fat and sweet tastes, respectively. LA was chosen because this polyunsaturated fatty acid, widely found in foods, binds with a high affinity the lipid receptors expressed in the taste buds (i.e., CD36 and GPR120) [24]. To reduce the potential nutritional interferences on the taste function due to food intake, all subjects were asked to eat the evening before the 3-AFC sessions, the following standardized meal: raw tomato (100 g) + 1 teaspoon of sunflower oil (5 g), bread (20 g of french baguette), chicken breast (120 g), potatoes cooked in water (250 g), green beans (100 g), butter (5 g), 1 yoghurt sweetened with cane sugar, 1 standard apple compote).

LA and sucrose thresholds were determined at 9 AM in fasting patients. In brief, subjects were asked to identify the sample that was different from the 2 others. Sets were presented in an ascending concentration from 0.00028% to 5% LA (wt/wt) spaced by 0.25 log units (18 solutions in total) and from 0.2% to 1.6% sucrose with an increment of 0.2% at each step (8 solutions in total). The procedure was stopped when the experimental sample was correctly identified 3 times, consecutively. This concentration represented the detection threshold value of the subject. Samples of 5 mL were tested at room temperature. Subjects were instructed to hold the sample in their mouth for 7 s, spit the solution out, and wait for 20 s before tasting the next sample. The interval between 2 sets was 60–120 s, during which participants were asked to rinse their mouth with water. To limit both visual and olfactory interferences, the samples were presented in opaque cups and participants wore a nose clip.

### 2.3. Preparation of Linoleic Acid Samples for 3-AFC Tests

The protocol used to prepare LA samples is fully detailed elsewhere [33]. In brief, LA (Sigma Aldrich) emulsions were prepared in a water solution containing 5% acacia gum (wt/wt; Merck), 5% mineral oil (wt/wt; Cooper), and 0.01% EDTA (wt/wt; VWR international). According to [32], acacia gum and mineral oil were added to limit viscosity and lubricity differences between control and experimental samples and EDTA was added to prevent the oxidation of LA. Samples were mixed conventionally by using a stirrer (Corning) and emulsified with a sonicator (Misonix sonicator model S-4000; QSonica LLC). The duration of the sonication was adapted to LA concentrations to obtain a similar particle size in order to minimize textural influences [33]. In all cases, sonication was conducted by lapses of 30 s separated by 1-min pauses. Sonication was conducted in a hermetic chamber saturated with nitrogen gas, using an ice bath and a temperature-controlled sonicator in order to limit the formation of oxidized compounds during the preparation of the emulsions.

### 2.4. Hormonal Assessments

Plasma insulin (LiaisonXL^®^, Diasorin, France), glucagon-like-peptide-1 (GLP-1) (EDI™ Total GLP-1 ELISA Kit—Epitope Diagnostics, Eurobio, France), peptide YY (PYY) and total ghrelin (U-PLEX Metabolic human assay, Meso Scale Discovery, Rockville, ML, USA) were assessed before (*t* = 0), 30 and 90 min after a test meal (Fresubin 2 kcal Drink^®^: 20 containing 400 Kcal, carbohydrates—35 g, lipids—20 g, protein—20 g). For GLP-1, blood collection was carried out in the presence of DPP4 inhibitor.

### 2.5. Statistics

Results are expressed as means ± SD. Data were analyzed with GraphPad Prism software (V7.01, Graphpad software Inc., San Diego, CA, USA) and sigmaplot. Thresholds data were analyzed with a paired Wilcoxon test with the Pratt’s method. Contingency data were analyzed with the X^2^-Fisher exact test. For the other data, after verification of the distribution normality they were analyzed using the Student’s t-test or the Mann–Whitney test with or without pairing. Correlations between the perception thresholds and hormonal concentrations were analyzed using Spearman’s correlations.

The principal component analysis (PCA) was carried out with R (v4.0.2; The R Foundation) software and the mixOmics package (v6.12.2) [34]. The purpose of this multivariate analysis was to explore whether the global changes in the satiety-regulating hormones from digestive tract elicited by surgery were sufficient to segregate patients who underwent VSGs from those who underwent RYGBs and to distinguish patients displaying a gustatory improvement from those not improved. In brief, the PCA was used to identify the principle sources of variations that are able to describe the different groups. The first component of the PCA explains most of the variability (*x*-axis), and the following components explain the remaining variability (*y*-axis).

### 2.6. Power Analysis

To our knowledge, the oral perception thresholds of lipid stimuli before and after VSG or RYGB surgery have never been explored before this study was undertaken. Therefore, our study size (i.e., ≥10 patients by surgery) was deduced from previously published data on the effects of VSG and RYGB on the perception thresholds of various tastants including sucrose [14,16]. This sample size seems to be a reasonable extrapolation especially due to the fact that each patient being under their own control leads to a reduction in the data variability.

## 3. Results

### 3.1. Changes of the Body Composition and Food Intake after VSG or RYGB

Similar decreases in the body mass and fat mass were found 6 months after surgery for both procedures (Table 1). Daily energy intake was also reduced to the same degree after the two types of surgery (−46.6% and 41.6% after VSG and RYBG, respectively). While the daily carbohydrate and protein intakes were similar in the surgery procedures, lipid consumption was slightly lower in the RYGB group than in the VSG patients (Table 2).

### 3.2. Impacts of VSG and RYGB on the Orosensory Perception of Lipid and Sweet Stimuli

**Figure 2:**
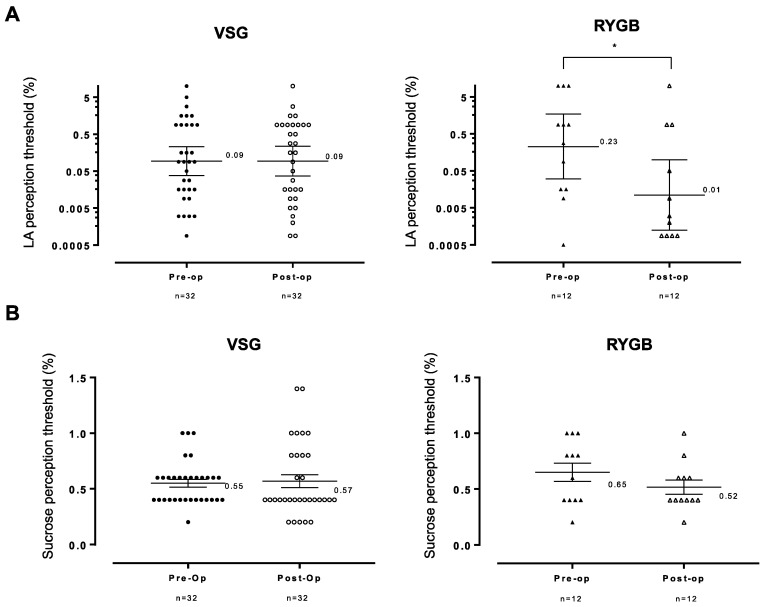
(**A**) Linoleic acid (LA) and (**B**) sucrose orosensory perception thresholds before and 6 months after vertical sleeve gastrectomy (VSG) or Roux-en-Y gastric bypass (RYGB) using the 3-alternative force-choice tests. Ascending concentrations of LA (from 0.00028% to 5% LA with 0.25 log units space, wt/wt) and sucrose (from 0.2% to 1.6% sucrose with increments of 0.2 g, wt/wt) were assessed. Geometric means, comparisons of perception thresholds before vs. after surgery were performed using a paired Wilcoxon–Pratt test. *, *p* < 0.05. Pre-op, preoperated; post-op, postoperated.

LA perception thresholds were characterized by a great interindividual variability at the origin of a large distribution covering four orders of magnitude (Figure 2A). In the VSG group, the mean value of LA thresholds did not differ before (preoperation) and after surgery (postoperation). By contrast, a significant decrease in the mean LA threshold was observed after RYGB suggesting that this surgery procedure might improve the orosensory sensitivity to LA stimuli (Figure 2A). For the sweet taste, the VSG procedure did not affect the mean perception threshold which was found to be 0.55% and 0.57% sucrose before and after surgery, respectively. A similar sucrose threshold distribution was also found in the RYGB group, despite a downward trend of mean perception values after surgery (0.65% sucrose in patients before RYGB vs. 0.52% after RYGB, *p* = 0.06—Figure 2B). No correlation between age and fat or sweet taste perception was found in these patients (age vs. LA perception thresholds, r = 0.003, *p* = 0.77; age vs. sucrose perception thresholds, *r* = 0.108, *p* = 0.77).

### 3.3. Changes in Subjective Food Sensations after VSG or RYGB

An improvement of taste sensations (i.e., sensitivity and intensity) was reported by most of the patients whatever the procedure used (Table 3). Subjective changes in orosensory perception (i.e., increased sensitivity) mainly concerned the sweetness, followed by the fattiness, and to a lesser extent the saltiness (Table 3). Although changes in bitterness perception were more rarely reported, their frequency was found to be slightly higher after RYGB than VSG (25% of patients vs. 3%, *p* = 0.056—Table 3). Whether a comparable change of fatty, salty and bitter taste intensity was reported by the two groups of patients, a more marked upward trend was observed for the sweet taste after RYGB (*p* = 0.07—Table 3). Postoperative impacts on olfactory performance were reported by less than half of patients (Table 3).

### 3.4. Impacts of Bariatric Surgery on the Fat and Sweet Tastes Are Not the Same in All Operated Patients

**Figure 3:**
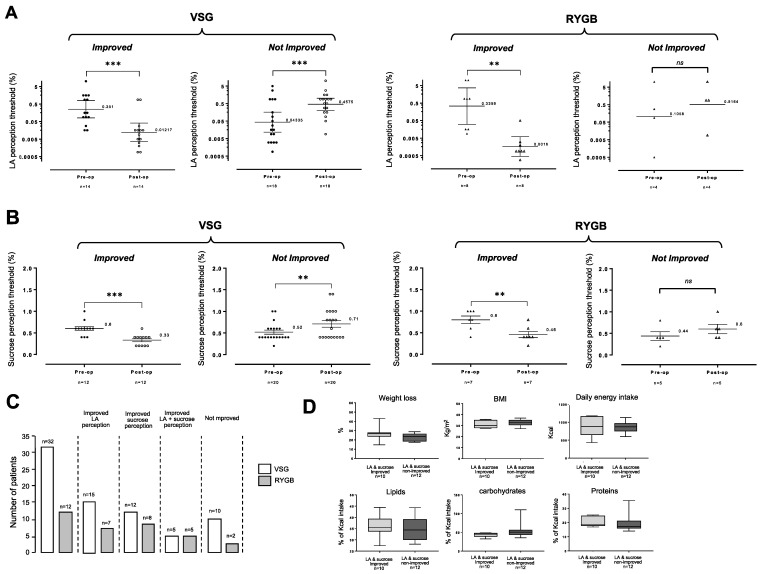
(**A**) Comparison of linoleic acid (LA) orosensory perception thresholds before and 6 months after vertical sleeve gastrectomy (VSG) or Roux-en-Y gastric bypass (RYGB) in improved and nonimproved patients. Ascending concentrations of LA (from 0.00028% to 5% LA with 0.25 log units space, wt/wt) were assessed. (**B**) Comparison of linoleic acid (LA) orosensory perception thresholds before and 6 months after vertical sleeve gastrectomy (VSG) or Roux-en-Y gastric bypass (RYGB) in improved and nonimproved patients. Ascending concentrations of sucrose (from 0.2% to 1.6% sucrose with increments of 0.2 g, wt/wt) were assessed. Geometric means, comparisons of perception thresholds before vs. after surgery were performed using a paired Wilcoxon–Pratt test. (**C**) Repartition of improved and nonimproved patients. (**D**) Post-operative comparison of weight loss, body mass index (BMI) and reported energy intake and composition using the four-day food recall in LA/sucrose-improved and nonimproved patients. **, *p* < 0.01; ***, *p* < 0.001; ns, non-significant.

To further explore the putative effects of bariatric surgery on the fatty and sweet tastes, LA and sucrose thresholds before and after surgery were compared in all patients. This investigation has allowed us to distinguish two subgroups whatever the surgery procedure used: patients displaying an improved sensitivity after surgery (i.e., lower thresholds) for fatty taste (Figure 3A) or sweet taste (Figure 3B) and nonimproved patients. Improvement of the orosensory perception of both LA and sucrose was found only in a limited number of subjects (i.e., VSG + RYBG, *n* = 10). This incidence seems to be greater after RYGB (5/12 patients, 41.6%) than after VSG (5/32 patients, 15.6%—Figure 3C). Complementary investigations are needed to validate this assumption. It is noteworthy that the double improved (i.e., LA and sucrose) patients (*n* = 10, Figure 3C) displayed a similar weight loss and pre- and postoperative BMI than nonimproved patients (*n* = 12, Figure 3C). These patients reported similar energy intakes and compositions (four-day food recall—Figure 3D).

### 3.5. Relationships between Appetite-Regulating Peptides and Sweet and Fatty Taste Perceptions after VSG or RYGB

**Figure 4:**
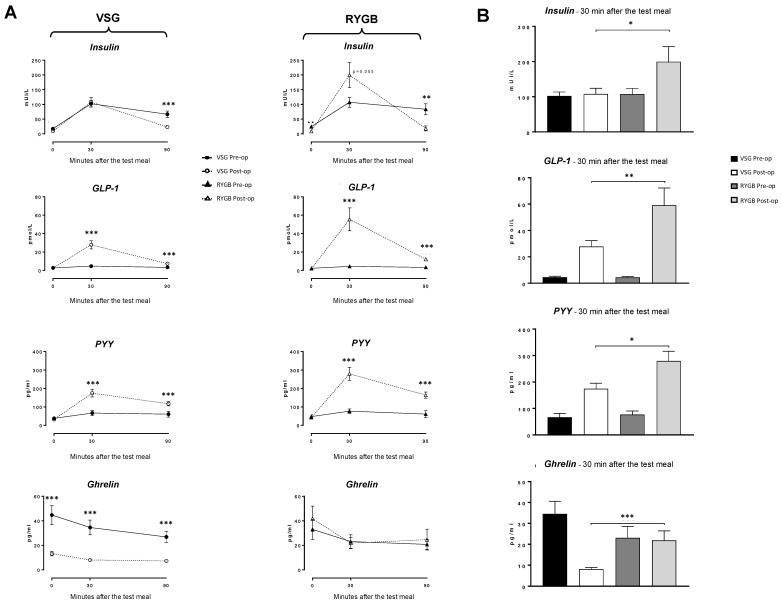
Plasma levels of appetite-regulating peptides following a test meal before (pre-op) and 6 months after surgery (post-op). (**A**) Kinetics of endocrine response. (**B**) Comparison of plasma endocrine levels 30 min (t30) after the test meal in patients who underwent a vertical sleeve gastrectomy (VSG) or a Roux-en-Y gastric bypass (RYGB). Glucagon-like peptide (GLP-1); peptide YY (PYY). *, *p* < 0.05; **, *p* < 0.01; ***, *p* < 0.001.

To explore whether appetite-regulating hormones might play a role in the improvement of the orosensory perception of fat and sugar, plasma insulin, GLP-1, PYY and ghrelin levels in response to a test meal were assayed before and after surgery. Meal-induced secretion of GLP1 and PYY increased after both surgeries (Figure 4A); this upregulation was significantly higher in the RYGB group. The ghrelin secretion was only affected by the VSG surgery (Figure 4B). In contrast to VSG, RYGB patients exhibited an improved insulin secretion after the test meal (Figure 4). The possible links between these changes in hormonal secretions and LA or sucrose perception thresholds were analyzed in the whole cohort by using Spearman correlations, but no significant association was found (see Table S1 in Supplemental Data).

Comparison of subjects with and without improvement of LA and sucrose perception thresholds did not show any significant difference in the hormonal responses to the test meal, except for insulin which showed a larger increase in patients displaying LA threshold improvement (Supplemental Data, Table S2).

**Figure 5:**
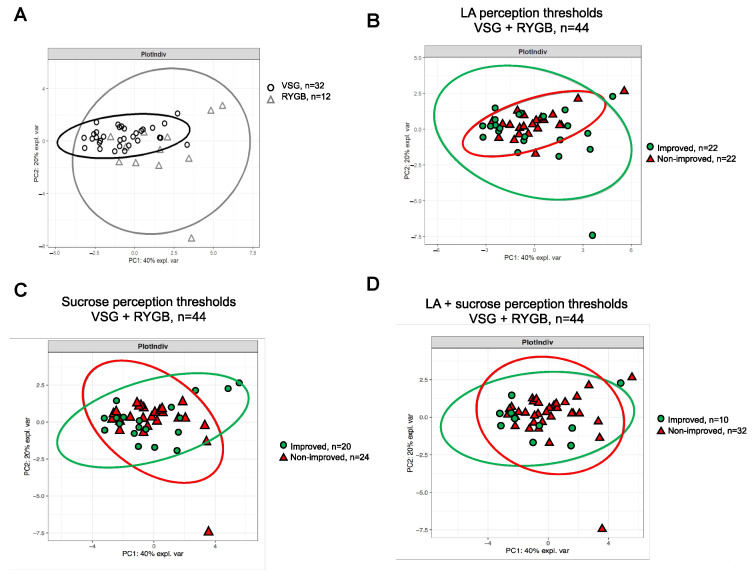
Principal component analysis (PCA) of gut hormones (i.e., insulin, GLP-1, PYY and Ghrelin) response to a test meal 6 months after surgery, as well as confidence ellipse analysis. (**A**) Comparison of VSG and RYGB patients. (**B**) Comparison of patients (VSG + RYGB) with and without improvement (improved vs. nonimproved) of the LA perception threshold. (**C**) Comparison of patients (VSG + RYGB) with and without improvement (improved vs. nonimproved) of the sucrose perception threshold. (**D**) Comparison of patients (VSG + RYGB) with and without improvement (improved vs. nonimproved) of the LA+ sucrose perception threshold.

Global analysis of these postingestive endocrine changes using the PCA method did not allow us to distinguish the patients who underwent a VSG from RYGB-operated patients (Figure 5A), suggesting that the improvement of fatty taste acuity found in RYGB patients is independent of this parameter. Therefore, both surgery groups (i.e., VSG + RYGB) were combined for further analysis. In spite of the strong difference of LA perception thresholds between patients displaying a fatty taste improvement after surgery and those not improved (Figure 3A), the PCA did not allow us to discriminate the two subgroups of patients (Figure 5B). A similar result was also found with sucrose alone (Figure 5C) or in combination with LA (Figure 5D).

## 4. Discussion

Whether the bariatric surgery may affect the fat and sweet taste sensitivity and thus contribute to the healthier food changes usually reported by the most of operated patients remains elusive. The present study was undertaken to explore further this issue. Data showed herein provide several new findings.

Firstly, RYGB leads to a significant decrease in LA perception thresholds (i.e., better fatty taste sensitivity) in contrast to VSG. Although this observation needs to be further confirmed by a study using a larger number of patients, these original data strongly suggest that the change of the oral fat perception after obesity surgery might be dependent on the procedure used. It is noteworthy that the magnitude of the weight loss does not explain the difference between the two surgeries—VSG and RYBG patients displayed similar BMIs and fat masses before and after surgery in our study. Although the impact of BMI as a variable in oral lipid sensitivity is a matter of debate in humans [24], this conclusion is consistent with the systematically reviewed literature [29]. A recent study using taste strip tests to evaluate the lipid detection thresholds in patients has reported an improvement of sensitivity to oleic acid stimuli 6 months after bariatric surgery including VSG [35]. This inconsistency with the present data might be due to olfactory inputs and patients not wearing nose clips in this study. Contrary to lipids, sucrose perception thresholds were similar in VSG and RYGB groups and remained unchanged after surgery. This result corroborates recent studies using similar psychophysical tests [15,16]. In contrast, an increased sweet taste sensitivity after surgery was mainly reported in strip test studies [19,20,21], suggesting that orosensory perception threshold assessments of lipid and sweet stimuli are highly dependent on the sensory methods used (3-AFC vs. strip test). Collectively, our data show that the RYGB procedure only affects the orosensory perception of lipids, suggesting that fat and sweet taste sensitivity might be differently regulated.

Secondly, using a direct measurement of the taste sensitivity, our data have highlighted that the impact of bariatric surgery on the fat and sweet tastes is not homogenous in all the patients, some displaying an improvement of sensitivity for LA and/or sucrose, while in others fat and sweet taste sensitivity remained unchanged or were even degraded. This disparity is reminiscent to what was already found for the fat taste in subjects with obesity [33]. The similar daily caloric intake and energetic nutrient distribution found in double-improved (i.e., LA and sucrose) and unimproved patients suggests a disconnection between the orosensory perception performance and eating behavior. Such a segregation between subjective food sensations and measured oral fat and sweet perception has already been found by others (for a review, [12]). Moreover, the present changes in food selection were evaluated using a self-reporting method (i.e., 4-day food recall questionnaire) known to produce biased responses. Indeed, patients with obesity tend to under-report their consumption of energetic-dense foods [36]. Whether the improvement of the fatty and sweet tastes found in some patients drives a healthier ingestive behavior remains to be further clarified using a direct method to accurately measure the changes in food selection (e.g., *ad libitum* buffet paradigm—[37]).

Thirdly, the changes in gut hormones observed in our study are in accordance with previous data [30,38]. A large body of evidence supports that the beneficial metabolic effects and the successful weight loss of RYGB and VSG are elicited by changes in the secretion of appetite-regulating peptides in response to a meal, especially insulin, GLP-1, PYY and ghrelin. In contrast to the well-documented remission of type-2 diabetes [39] and the promotion of satiety [40], effects of these endocrine changes on the gustatory function remains elusive [41]. Indeed, they are mainly supported by indirect observations frequently derived from animal models: presence of receptors from appetite-regulating hormones in taste bud cells, endocrine modulation of taste perception, self-reported improvement of taste sensations (sensitivity and intensity) after surgery. Using a multivariate statistical method (PCA), we have found that this new hormonal environment is insufficient for the discrimination of patients with improved orosensory perceptions of LA and/or sucrose after surgery from nonimproved patients. This finding strongly suggests that the gustatory regulation by hormones from the digestive tract, if it exists in humans, has a very limited impact on this parameter. The fact that taste bud cells express appetite-regulatory peptides and their cognate receptors suggests a local (i.e., at the level of the gustatory papillae) rather than a peripheral (i.e., at the level of the digestive tract) endocrine control of the taste function.

Finally, according to previous studies (for a review see, [12]), we found that subjective changes in food sensations reported by the most of patients after surgery (i.e., increased sensitivity and intensity to fat and sweet stimuli) were not corroborated by the direct measure of taste perception using a validated method, excepted for fat after RYGB. This lack of a causal link suggests that self-reported changes might result from other mechanisms such as, for example, a modification of rewarding value of foods [24].

The main weakness of our study is the low number of patients included in the RYGB group (*n* = 12) as compared to VSG group (*n* = 32). This imbalance reflects the current tendency to favor the VSG procedure by reason of reduced postsurgery complications [42]. Despite this limitation, it is noteworthy that our study reproduces the lack of change in the sucrose perception thresholds found with a greater number of RYGB patients (n = 23) [16]. Moreover, the fact that investigations were performed only in women who were their own controls has likely reduced the possible biases due to gender and interindividual variability [25]. Finally, to optimize the reproducibility of psychophysical data, the 3-AFC tests were performed in the morning by the same experimenter in fasted patients who ate the same standardized meal the night before.

In conclusion, despite that the most of the patients, regardless of the surgery procedure, self-reported a better orosensory perception of fattiness and sweetness, improvement of fat and sweet taste perceptions was only found for a few patients, with the exception of RYGB patients being predominantly more sensitive to the taste of fat after surgery. The lack of evident implications of appetite regulatory hormones in these surgery-mediated orosensory changes emphasizes the complexity of mechanistic events linking obesity, and conversely bariatric surgery, to perceived food sensations. Whether the improvement of fat after the RYGB procedure provides physiological advantages to these patients contributing, for example, to the long-term success of surgery, remains to be explored. Understanding of the underlying mechanisms might be useful to segregate improved from nonimproved patients in the future in order to offer them early personalized therapies promoting long-term weight loss.

## Figures and Tables

**Table 1:** Body composition before *(pre-op)* and 6 months *(post-op)* after surgery.

Variables	VSG (*n* = 32)	*p*-Value Pre-op vs. Post-op	RYGB (*n*=12)	*p*-Value Pre-op vs. Post-op	*p*-Value Post-op (VSG vs. RYGB)
Body mass (kg)					
*Pre-op*	115.8 ± 2.3		111.7 ± 3.8		*ns*
*Post-op*	85.2 ± 2.1	***	85.4 ± 3.0	***	*ns*
*Weight loss (kg)*	30.5 ± 1.2		26.2 ± 1.7		0.06
*Weight loss (%)*	26.4 ± 1.0		23.3 ± 1.2		*ns*
BMI (kg/m^2^)					
*Pre-op*	43.1 ± 0.7		42.3 ± 1.0		*ns*
*Post-op*	31.6 ± 0.6	***	32.1 ± 0.9	***	*ns*
Fat mass (% BM)					
*Pre-op*	51.6 ± 0.5		51.2 ± 0.9		*ns*
*Post-op*	43.3 ± 1.0	***	43.9 ± 1.2	***	*ns*

Means ± SD; ***, *p* < 0.001; ns, not significant; Pre-op vs. Post-op, paired Student; VSG vs. RYGB, Student. Op, operated.

**Table 2:** Reported food intake (4-days food recall) before *(pre-op)* and 6 months *(post-op)* after surgery.

Variables	VSG (*n* = 32)	*p*-Value Pre-op vs. Post-op	RYGB (*n* = 12)	*p*-Value Pre-op vs. Post-op	*p*-Value Post-op (vsg vs. rygb)
Food intake *(g/d)*					
*Pre-op*	1173.0 ± 39.9		1083.0 ± 90.8		*
*Post-op*	568.7 ± 35.5	***	707.3 ± 64.4	**	
Energy intake *(Kcal/d)*					
*Pre-op*	1564.5 ± 48.8		1557.3 ± 90.1		*ns*
*Post-op*	835.5 ± 44.3	***	908.2 ± 61.0	***	
Carbohydrates (% energy intake)					
*Pre-op*	46.1 ± 1.0		48.0 ± 1.2		*ns*
*Post-op*	44.0 ± 1.5	*ns*	47.7 ± 2.4	*ns*	
Lipids (% energy intake)					
*Pre-op*	34.0 ± 0.9		31.8 ± 1.3		*
*Post-op*	37.4 ± 3.1	*	32.6 ± 1.9	*ns*	
Proteins (% energy intake)					
*Pre-op*	20.0 ± 0.6		20.1 ± 0.7		*ns*
*Post-op*	18.8 + 0.6	*ns*	19.6 ± 1.0	*ns*	

Means ± SD; *, *p* < 0.05; **, *p* < 0.01; ***, *p* < 0.001; ns, not significant; Pre-op vs. post-op, paired Student; VSG vs. RYGB, Student. Op, operated.

**Table 3:** Reported gustatory and olfactory changes (TOC questionnaire) 6 months after surgery.

	VSG (*n* = 32)	RYGB (*n* = 12)	*p*
**Taste changes**			
Increased taste (% of patients)	56	75	*ns*
*Intensity of change (1–10)*	6.4 ± 2.0	6.0 ± 1.1	*ns*
**Increased sweet taste (% of patients)**	78	75	*ns*
*Intensity of change (1–10)*	4.9 ± 3,1	6.9 ± 1.6	*0.071*
**Increased fat taste (% of patients)**	69	67	*ns*
*Intensity of change (1–10)*	6.4 ± 2.7	7.8 ± 1.8	*ns*
**Increased salt taste (% of patients**)	50	58	*ns*
*Intensity of change (1–10)*	5.6 ± 2.4	5.0 ± 2.4	*ns*
**Increased bitter taste (% of patients)**	3	25	*0.056*
*Intensity of change (1–10)*	6.0 ± 1.6	6.3 ± 1.2	*ns*
**Olfactory change (% of patients)**	50	33	*ns*
Increased olfaction (% of patients)	41	25	*ns*
*Intensity of change (1–10)*	5.7 ± 1.8	4.3 ± 1.2	*ns*

Means ± SD; ns, not significant; *p* = X^2^ test of Fisher exact test.

## Data Availability

The data presented in this study are available on request from the corresponding author. The data are not publicly available due to ethical restrictions.

## Supplementary Materials

The following are available online at https://www.mdpi.com/2072-6643/13/3/878/s1, Table S1: Relationships between appetite--regulating hormones changes after a test meal and LA or sucrose perception thresholds in the whole cohort (VSG + RYGB) before (pre--op) and after (post--op) surgery, Table S2. Appetite-regulating hormones changes in subjects with improved or not improved fat and/or sweet taste perception thresholds.
