# Supplementary material for: Orosensory Perception of Fat/Sweet Stimuli and Appetite-Regulating Peptides before and after Sleeve Gastrectomy or Gastric Bypass in Adult Women with Obesity
Source: Nutrients. 2021 Mar 8;13(3):878. doi: 10.3390/nu13030878 (PMC8000537; doi:10.3390/nu13030878)
Supplement: Supplementary file 1 [file nutrients-13-00878-s001.pdf]

Table 1-S. Relationships between appetite-regulating hormones changes after a test meal and LA or sucrose perception thresholds in the whole cohort (VSG+RYGB) before (pre-op) and after (post-op) surgery

	**Pre-op LA thresholds**		**Pre-op sucrose thresholds**	
	R	p	R	p
t-AUC GLP-1	0.112	0.439	-0.071	0.651
t-AUC Insulin	-0.083	0.595	-0.138	0.376
t-AUC PYY	0.285	0.074	-0.183	0.257
t-AUC Ghrelin	-0.020	0.901	-0.275	0.086
t-30 GLP-1	0.103	0.515	-0.102	0.519
t-30 Insulin	-0.103	0.509	-0.077	0.620
t-30 PYY	0.307	0.054	-0.104	0.522
t-30 Ghrelin	0.005	0.976	-0.282	0.077
	**Post-op LA thresholds**		**Post-op sucrose thresholds**	
	R	p	R	p
t-AUC GLP-1	0.116	0.486	0.317	0.052
t-AUC Insulin	-0.108	0.504	0.216	0.180
t-AUC PYY	0.111	0.493	0.094	0.562
t-AUC Ghrelin	0.020	0.905	0.025	0.879
t-30 GLP-1	0.104	0.526	0.334	0.037
t-30 Insulin	-0.146	0.355	0.234	0.135
t-30 PYY	0.087	0.619	0.099	0.543
t-30 Ghrelin	0.064	0.626	0.060	0.717

Spearman correlations. LA, Linoleic acid; t-AUC, total area under the curve; t-30, peak value 30 minutes after the test meal; GLP-1, glucagon like peptide 1; PYY, peptide YY.

Table 2-S. Appetite-regulating hormones changes in subjects with improved or not improved fat and/or sweet taste perception thresholds.

Whole cohort (VSG+RYGB)

LA thresholds	Improved (n =22)	Not improved (n =22)	p
GLP-1 AUC ratio	7.96 ± 10,79	8.89 ± 9.96	0.7931
Insulin AUC ratio	1.35 ± 0.67	0.85 ± 0.49	0.0112
PYY AUC ratio	3.06 ± 1.80	3.58 ± 2.17	0.4368
Ghrelin AUC ratio	0.59 ± 0.53	0.90 ± 1.33	0.3841
Sucrose thresholds	Improved (n = 20)	Not improved (n =24)	p
GLP-1 AUC ratio	10.08 ±12.52	7.41 ± 8.72	0.4481
Insulin AUC ratio	1.06 ± 0.67	1.12 ± 0.60	0.7970
PYY AUC ratio	3.32 ± 2.17	3.36 ± 1.93	0.9527
Ghrelin AUC ratio	0.72 ± 0.49	0.79 ± 1.30	0.8389

RYGB

LA threshold	Improved (n =7)	Not improved (n =5)	p
GLP-1 AUC ratio	12.99 ± 15.89	11.53 ± 10.69	0.8745
Insulin AUC ratio	1.71 ± 0.66	0.90 ± 0.80	0.1011
PYY AUC ratio	3.20 ± 1.69	5.90 ± 3.28	0.0992
Ghrelin AUC ratio	1.11 ± 0.38	2.87 ± 2.27	0.0671
S threshold	Improved (n = 6)	Not improved (n =6)	p
GLP-1 AUC ratio	14.42 ± 16.81	10.11 ± 10.60	0.6282
Insulin AUC ratio	1.53 ± 0.85	11.29 ± 0.78	0.6311
PYY AUC ratio	3.82 ± 2.75	4.61 ± 2.67	0.6453
Ghrelin AUC ratio	1.19 ± 0.40	2.42 ± 0.2.19	0.2049

VSG

LA threshold	Improved (n =15)	Not improved (n =17)	p
GLP-1 AUC ratio	4.75 ± 4.16	8.14 ± 10.03	0.3027
Insulin AUC ratio	1.14 ± 0.60	0.84 ± 0.41	0.1319
PYY AUC ratio	2.96 ± 1.95	3.03 ± 1.48	0.9208
Ghrelin AUC ratio	0.23 ± 0.21	0.43 ± 0.23	0.0304
S threshold	Improved (n = 12)	Not improved (n =20)	p
GLP-1 AUC ratio	6.83 ± 8.26	6.65 ± 8.32	0.9603
Insulin AUC ratio	0.81 ± 0.40	1.07 ± 0.56	0.1969
PYY AUC ratio	2.98 ± 1.80	3.01 ± 1.60	0.9656
Ghrelin AUC ratio	0.40 ± 0.22	0..34 ± 0.25	0.1996

Changes in the hormonal response to the test meal are expressed as the ratio of post-operative to pre-operative values of area under the curve (AUC). Means±SD. LA, linoleic acid; GLP-1, glucagon like peptide 1; PYY, peptide YY.
